# Continuous-Flow Production of Injectable Liposomes via a Microfluidic Approach

**DOI:** 10.3390/ma10121411

**Published:** 2017-12-10

**Authors:** Alessandra Zizzari, Monica Bianco, Luigi Carbone, Elisabetta Perrone, Francesco Amato, Giuseppe Maruccio, Filippo Rendina, Valentina Arima

**Affiliations:** 1 CNR NANOTEC-Institute of Nanotechnology, c/o Campus Ecotekne, University of Salento, via Monteroni, 73100 Lecce, Italy; alessandra.zizzari@unisalento.it (A.Z.); monica.bianco@nanotec.cnr.it (M.B.); luigi.carbone@nanotec.cnr.it (L.C.); elisabetta.perrone@nanotec.cnr.it (E.P.); francesco.amato@nanotec.cnr.it (F.A.); giuseppe.maruccio@unisalento.it (G.M.); 2 Department of Mathematics and Physics “E. De Giorgi”, University of Salento, via Arnesano, 73100 Lecce, Italy; 3 Janssen Pharmaceutical Company of Johnson & Johnson, via C. Janssen, Borgo S. Michele, 04100 Latina, Italy; FRENDINA@its.jnj.com

**Keywords:** liposomes, microfluidics, microreactors

## Abstract

Injectable liposomes are characterized by a suitable size and unique lipid mixtures, which require time-consuming and nonstraightforward production processes. The complexity of the manufacturing methods may affect liposome solubility, the phase transition temperatures of the membranes, the average particle size, and the associated particle size distribution, with a possible impact on the drug encapsulation and release. By leveraging the precise steady-state control over the mixing of miscible liquids and a highly efficient heat transfer, microfluidic technology has proved to be an effective and direct methodology to produce liposomes. This approach results particularly efficient in reducing the number of the sizing steps, when compared to standard industrial methods. Here, Microfluidic Hydrodynamic Focusing chips were produced and used to form liposomes upon tuning experimental parameters such as lipids concentration and Flow-Rate-Ratios (FRRs). Although modelling evidenced the dependence of the laminar flow on the geometric constraints and the FRR conditions, for the specific formulation investigated in this study, the lipids concentration was identified as the primary factor influencing the size of the liposomes and their polydispersity index. This was attributed to a predominance of the bending elasticity modulus over the vesiculation index in the lipid mixture used. Eventually, liposomes of injectable size were produced using microfluidic one-pot synthesis in continuous flow.

## 1. Introduction

Liposome vesicles with 50–250 nm size represent very suitable vector systems for targeted drug delivery [1,2]. On one hand, a sphere-shaped membrane bilayer, normally made of nontoxic phospholipids, creates a confined aqueous habitat within which hydrophilic bioactive cargos may be loaded. On the other hand, lipophilic molecules or long alkyl chain-surrounded nanoparticles may be entrapped within the hydrophobic compartment of the lipid membrane [3]. For use as biomedical formulations, the steric stabilization of liposomes is typically addressed via surface functionalization of phospholipids, with the purpose to prolong their blood circulation time and circumvent their possible capture by the mononuclear phagocytic system [1,4]. For instance, polyethylene glycol (PEG) chains grafted on the liposome surface were employed to increase their relatively low stability in vitro and to prevent their immediate uptake and clearance by the reticuloendothelial system in vivo [5,6]. However, the use of long-chain PEG precursors in liposome formulations leads to operative complications during synthesis by introducing solubility restrictions, influencing the transitions temperature of the membranes and, hence, the ultimate size of liposomes [7].

In many conventional nanoscale liposome synthesis methods, the control over the parameters which determine the formation of vesicular systems (i.e., injection flow velocity, injection pressure, temperature, and stirring rate) is limited by the chaotic nature of the reagents mixing under turbulent conditions and obscured by the visual inaccessibility of the mixing dynamics. By contrast, thanks to a precise control over mixing and interdiffusion under laminar flow conditions and to a highly efficient heat transfer, the microfluidic technology permits a continuous in-flow formation of size-monodisperse nanovesicles [8,9,10] with a negligible need for post-processing procedures of size homogenization (i.e., membrane extrusion or sonication). This determines important benefits for personalized medicine applications such as drug delivery and gene therapy, which require reproducible vesicle size distributions and consistency from batch to batch.

In this context, Microfluidic Hydrodynamic Focusing (MHF) represents the simplest and most promising method for nanoscale liposome formation [9,10]. A typical MHF system employs a mixing microchannel with three-branched inputs where an aqueous solution flows along the two lateral paths, while an alcoholic solution of membrane lipids (like cholesterol and a variety of phospholipids) is injected along the central branch. The limited duct dimension provides that the pressure-driven flows remain stratified inside the mixing channel. At this stage, as the aqueous solution diffuses into the lipid stream and dilutes the alcohol domain, the polarity of the lipid system increases, lipids become progressively less soluble and, therefore, tend to assemble spontaneously into spherical vesicles [11]. The average diameter of MHF-produced nanovesicles is affected by the phospholipid intrinsic features (charge and phase transition temperature) as well as by the chip architecture (inlet geometry, mixing microchannel cross section, etc.), the flow parameters (the ratio of the aqueous buffer to the alcoholic solution flow rate, namely, FRR, and the total volumetric flow rate, namely, Q_t_), the mixing temperature, and the lipids concentration. As compared to the “controlled ethanol injection” method, at set conditions of lipids concentration in alcohol and of water-to-alcohol molar ratio, liposomes produced using MHF appeared smaller and more monodisperse in size without any influence on size stability over time [10]. In addition, changes of the hydrodynamic boundary conditions enhance MHF flexibility in targeting liposomes of specific particle size.

In the view of industrial applications of the present method, the MHF approach was efficiently translated to a larger scale using the vertical flow focusing (VFF) concept [12].

In this work, we adapted the MHF technology to a well-established pharmaceutical industrial process to obtain, on a laboratory scale and in a single step, the liposomes used in the well-known commercial product Doxil (R). After production, the liposomes can be loaded with doxorubicin hydrochloride with high yields using an ammonium sulphate gradient [13,14]. 

The adjustment of the microfluidic process resulted not straightforward considering the operative restrictions that pharmaceutical companies introduced in relation to: *(i)* the obligation imposed upon the use of selected lipid compositions and specifications (as covered by international patents and registration files submitted to Health Authority); *(ii)* the commercial convenience to use low-cost and easily available raw materials. In fact, this lipid composition consists of hydrogenated soy phosphatidylcholine (HSPC), *N*-(carbonyl-methoxypolyethylene glycol 2000)-1,2-distearoyl-sn-glycero-3-phosphoethanolamine (mPEG2000-DSPE), and cholesterol (CHOL) in 56.2/38.5/5.3 mol/mol. HSPC is essentially equivalent to 1,2-distearoyl-sn-glycero-3-phosphocholine (DSPC), (it contains about 85% of DSPC and 15% of dipalmitoylphosphatidylcholine (DPPC)), but with lower cost and higher availability of GMP (good manufacturing practices) quality raw materials. However, because of its lower solubility in ethanol (preferred to isopropanol or other organic solvents for toxicity reasons) [15] and its higher transition temperature, its presence in the lipids mixture requires injection and manipulation at temperatures above 50 °C. Furthermore, the process is carried out in the presence of a high concentration of saline species to allow the loading of the drug, and this can cause clogging of the microchannels. 

In order to produce liposomes suitable for parenteral administration (size below 100 nm and polydispersity index ≤ 0.2), the liposome size dependence at different FRRs versus injection orientation and lipids concentration was investigated at predefined fixed temperature and lipids ratio. 

The study was supported by the modelling of the laminar flow dependence upon geometric constraints and FRR conditions. 

## 2. Results

Here we will first focus on the rationale for the design of the microfluidic chips and of the experimental set up (Section 2.1), and then on the variables involved in liposome formation (Section 2.2, Section 2.3 and Section 2.4).

### 2.1. Design of the Microfluidic Set Up

**Figure 1:**
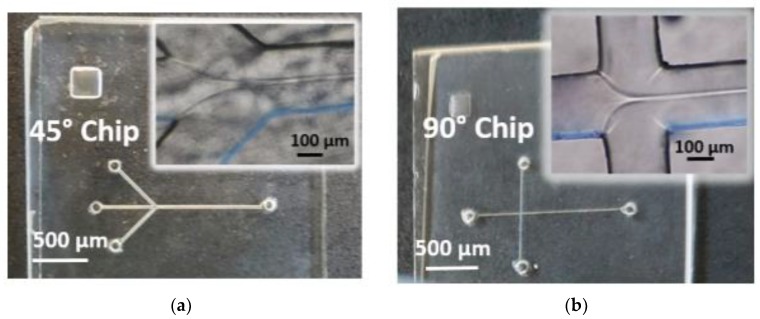
Images of (**a**) 45° chips and (**b**) 90° chips with zoomed images in the focusing regions shown in the insets. The ethanol laminas formed at Flow-Rate-Ratios FRR = 10 and total volumetric flow rate Q_t_ = 105 μL/min in the 45° chip, and FRR = 2, and Q_t_ = 90 μL/min in the 90° chip.

Glass microreactors are robust and multiuse chips that can be fabricated using low-cost techniques such as optical lithography, chemical wet etching, and thermal bonding [16,17,18]. In this study, two different inlet geometries were investigated, as shown in Figure 1. Moreover, from the same masks and by exploiting the isotropic chemical etching of the glass substrates, microreactors of different internal volumes were produced and labeled as “Small”, S, “Medium”, M, and “Large”, L. Specifically, semicircular cross sections mixing channels (see Scheme S1) were fabricated with height h and width w (see Table S1) dependent on the nominal thickness and geometry. A fine control of the microchannel size is the first requirement to generate a highly focused stream of lipids and to obtain narrow size distributions thereof. Liposomes were synthesized by injecting an ethanol-based lipid mixture from the left in the center channel while injecting a (NH_4_)_2_SO_4_ aqueous solution into the two oblique (or perpendicular in the case of the 90° geometry) side channels. 

Five chips labelled as “S” (with h ≈ 25 μm) and “M” (with h ≈ 50 μm) were tested at a low lipid concentration (0.9 mg/mL); however, clogging was experienced as soon as the lipid solution met the aqueous stream. The precipitation of solid material was observed in the mixing channels of all devices (independently of the set FRR) at the beginning of the experiments before reaching a stabilization of the flow conditions. In order to avoid clogging, the synchronization of the aqueous and ethanol flow streams was attempted, but was unsuccessful. The poor solubility of the lipids when forming the liposome bilayers in the “S” and “M” chips forced us to stop additional investigations. On the contrary, the experiments performed using the “L” chips (h ≈ 100 μm) were successful as the fluid streams easily cleaned the microreactor channel, reaching a reliable steady state. 

In the “L” chips, different flow conditions and total lipid concentrations were tested (as reported in the Section 4), keeping the temperature constant. The temperature of 75 °C set on the microscope stage where the chip was located allowed the mixing of lipid streams above the phase transition temperature. The heating of the lipids inside the syringe using a heating tape set at 55 °C during the injection was used to overcome solubility issues (see Figure S1).

The samples obtained from the experiments with such “L” chips were analyzed via dynamic light scattering (DLS) and, in some cases, also by transmission electron microscopy (TEM).

In order to describe the controlled mechanical conditions of the microfluidic process [19], the laminar flow dependence on geometric constraints at different FRR conditions was studied by comparing the experimental results with numerical simulations. In addition, the effects of the geometric constraints and the lipid concentrations on the liposomes size at different FRRs were explored.

### 2.2. Liposome Formation: The Influence of the Ethanol Stream Width 

As a first step, an analysis of the possible experimental flow conditions to be used in the 45° and 90° chips and of the dimensions of the focused ethanol stream was performed and compared with numerical simulations (see Section 4).

From preliminary experiments, it was evaluated that the 45° chips could be tested within a wide range of FRR values (up to 100), whereas the 90° chips were tested only up to FRR = 6, as a return flux of the lateral aqueous streams inside the central inlet occurred at higher FRRs. Thus, to evaluate the microreactor geometry efficacy towards liposome synthesis, FRR was set to 5, and the lamina behavior along the mixing channel length was analyzed in both chips.

**Figure 2:**
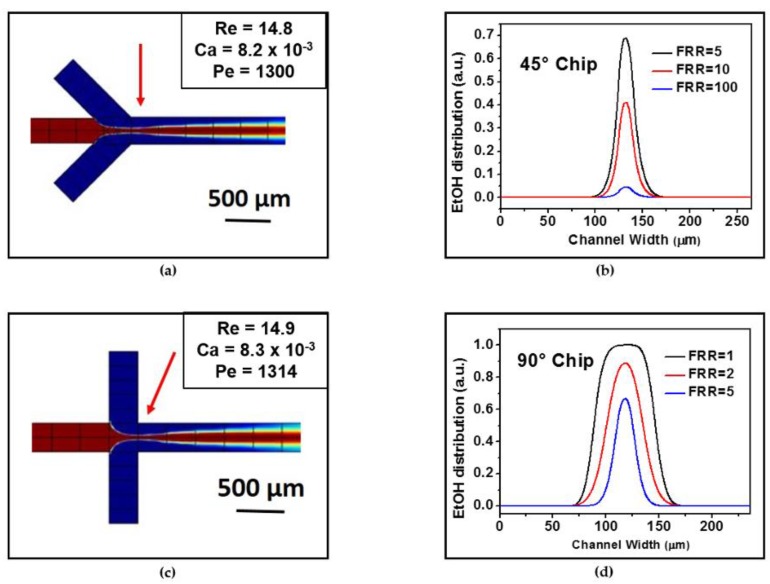
The colour maps represent the distribution of ethanol (EtOH) in the hydrodynamic flow focusing region for (**a**) 45° chips and (**c**) 90° chips at a FRR = 5, and Q_t_ of 30 µL/min and 150 µL/min. The red and blue colors refer to the EtOH and water domains, respectively. The maps reproduce the mixing of the two fluids occurring in the mixing channel. The red arrows indicate the mixing channel position where the ethanol distributions (shown in (**b**,**d**)) and the values of Re, Pe, and Ca (insets of Figure 2a,c) were calculated. The values of the peak areas estimated at three different FRRs are reported in Table 1.

Figure 2a,c show the continuous-flow mixing kinetics of the reagent streams in both microfluidic chip geometries. Notably, in the 90° architecture the ethanol stream appeared more hydrodynamically focused than in the 45° architecture in the region at the beginning of the mixing channel (indicated by the red arrow) because of the higher shear forces generated by the two lateral aqueous streams. The ethanol stream focusing was associated to slightly higher Reynolds (Re), Capillary (Ca), and Péclet (Pe) numbers in the 90° chips (see inset in Figure 2c). Subsequently, a faster interdiffusion with the water domains occurred along the mixing channel length, as evidenced by the dilatation of the laminar interface. The 45° chips, instead, displayed a slightly less focused organic stream and relatively smaller Re, Pe, and Ca numbers at the entrance of the mixing channel. This caused a slower interface diffusion, which extended the lamina far beyond the channel length without a significant broadening. In addition to the color maps showing the concentration gradient, this was evidenced in the ethanol distributions plotted in Figure S2. The distributions were calculated along the width of the mixing channel (in the point indicated by the red arrows in Figure 2a,c) and were proportional to the dimensions of the ethanol/lipid lamina in the experiments.

The simulated distributions reported in Figure 2b,d show how the ethanol amount gradually decreased as the FRRs increased for the 45° and the 90° chips. This corresponded to a reduction of the ethanol lamina stream at higher FRRs.

We then correlated the dimensions of the experimental stream width with the simulated ones using the areas of the two peaks of Figure S2 as parameters. The values reported in Table 1 demonstrate that, on the assumption of the same FFR conditions, the organic lamina appeared larger for the 45° chips than for the 90° chips. These are in qualitative agreement with the ethanol stream widths experimentally determined, namely, 21 ± 2 μm for the 45° chips and of 5 ± 3 μm for the 90° chips (see the inset in Figure 1 and the data reported in Table 1).

To estimate the FRR effect for the same chip geometry, the areas of the peaks of Figure 2b,d were calculated (after verifying that they appeared to be dependent on FRR and not on Q_t_, see Figure S4, as expected at high focusing conditions [20]) and compared with the experimentally estimated lamina dimensions (see Table 1). A good agreement was observed for the 90° chips as both the experimental stream widths and the areas of the peaks roughly halved their values from FRR 1 to 2 and up to 5. The theoretical trend was also respected in the case of the 45° chips, but with higher accuracy at low FRRs since both the experimental stream widths and the areas of the peaks roughly halved their values from FRR 5 to 10. Less agreement was observed at the high FRR = 100, since the peak area was reduced by one order of magnitude, but the experimental lamina was roughly 1/3. The mismatch between the calculated and the experimental values at a high FRR could be due to the poor resolution of the thin lamina, as visualized by optical microscopy in the presence of solutes, which act as surfactants.

### 2.3. Liposome Formation: Effect of the Geometric Constraints

**Figure 3:**
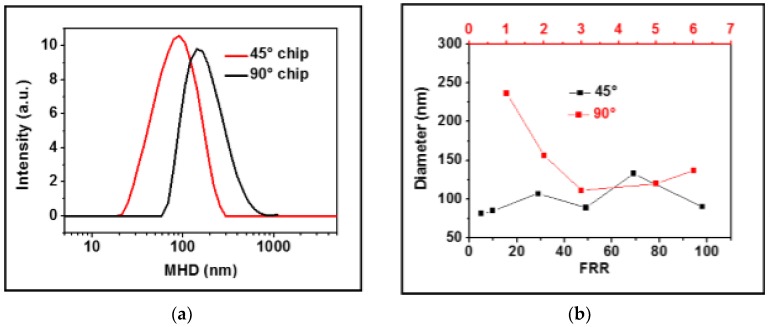
(**a**) Dynamic light scattering (DLS) spectra of liposome solutions produced inside 45° and 90° chips at FRR = 5 and at a lipid concentration of 0.9 mg/mL; *(***b**) plot of the Z-average mean hydrodynamic diameter (MHD) versus the FRR of liposomes produced at a lipid concentration of 0.9 mg/mL inside 45° and 90° chips. The black x axis is related to FRRs used in the 45° chips and the red one (upper side of graph) to FRRs used in the 90° chips.

Possible influences of the inlet geometries on the Mean Hydrodynamic Diameter (MHD) and the polydispersity index (PDI, which represents the square of the ratio between the absolute width and the mean of the DLS spectra distributions) of the liposomes were studied. The direct comparison between the two 45° and 90° chips was performed at FRR = 5 and at the lowest lipid concentration. As observed in Figure 3a, liposome Z-average MHD was of 92 nm with a wide PDI (0.35) for the 45° chips, while in the case of the 90° chips, the average size was apparently larger (120 nm), but a with lower PDI (0.27). 

The MHD of the main peaks, as determined from the DLS spectra of the samples produced at a lipid concentration of 0.9 mg/mL and at different FRRs using the 90° chip, are reported in Table S2. The values show that larger liposomes were produced at FRR ≤ 2.

Figure 3b represents a comparison between the Z-average MHD of the liposomes produced with the two chips at different FRRs. It is noteworthy that while the 90° chips provided larger liposomes at low FRRs and then stabilized reaching a plateau, the 45° chips did not show a clear correlation between the FRRs and the average particle size.

### 2.4. Liposome Formation: Effect of Lipids Concentration

Based on the requirement of producing injectable liposomes of targeted size, the lipids concentration was investigated using a 45° geometry-based microreactor. All the formulations were analyzed by DLS to determine the MHD and PDI of the main peak.

**Figure 4:**
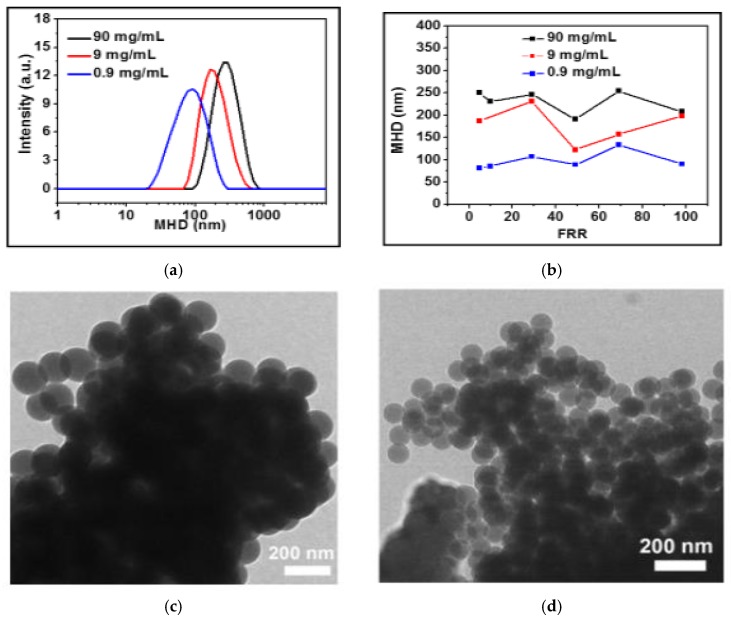
(**a**) DLS spectra of liposome solutions produced inside 45° chips at FRR = 5, at three different lipids concentrations; (**b**) plot of the Z-average MHD versus the FRR at three different lipids concentrations inside 45° chips. Transmission electron microscopy (TEM) pictures of vesicles obtained through a 45° chip; (**c**) at FRR = 30 and lipids concentration of 90 mg/mL; and (**d**) at FRR = 70 and lipids concentration 0.9 mg/mL.

As shown in Figure 4a, a decrease in the lipids concentration of one or even two orders of magnitude caused a shift of the size distribution to lower values; bimodal size distributions, which could be occasionally observed at high concentration (90 mg/mL), disappeared as the lipids concentration decreased.

To compare the results in the presence of two peaks, the Z-average MHD, as calculated by DLS, was adopted. The MHD values of the main peaks, produced at different lipids concentration and FRRs, are reported in Table S3. In detail, the sizes determined at different lipids concentrations were respectively: 190 ÷ 255 nm (PDI, 0.26 ÷ 0.50) at 90 mg/mL; 122 ÷ 230 nm (PDI, 0.21 ÷ 0.37) at 9 mg/mL; 80 ÷ 130 nm (PDI, 0.18 ÷ 0.35) at 0.9 mg/mL (see Figure S5).

As illustrated in Figure 4b, no monotonic decrease in liposome MHD was observed at increasing FRRs, suggesting that the lipids concentration is a more critical parameter than the FRRs for the characteristics and mixture of the employed lipids.

The dependence of liposome size on lipids concentration was also confirmed by TEM analysis. Figure 4c–d report a few examples of nanosized liposome vesicles obtained at 90 mg/mL, and 0.9 mg/mL, respectively. The vesicles exhibited a size as large as 130 ± 9 nm at higher values of concentration, and 78 ± 5 nm at lower values, as determined by measuring almost 200 nanoparticles. The differences with respect to the dimensions of the same samples estimated via DLS (i.e., 381 ± 295 nm and 104 ± 63 nm) were interpreted in light of the dehydration event that liposome solutions undergo (see Section 4) and which can alter the final vesicle size. However, this interpretation, which could justify the difference between large and small particle results obtained by DLS and TEM, and the bigger impact with large vesicles, needs further investigation. As a general consideration, all tested samples showed monodisperse vesicle sizes and shapes as well as a tendency to aggregation when deposited onto a solid substrate. This can be mainly interpreted as a consequence of *(i)* the lack of surface charge of the liposomes (ζ-potential almost 0 mV); and *(ii)* the water evaporation after the deposition of the vesicle solution onto a support, which concentrates the nanoliposomes in increasingly smaller circular domains.

To confirm that the spherical structures observed in TEM images were liposomes, a microdifferential scanning calorimetry (mDSC) was performed. We observed that mDSC traces showed a broad endothermic transition in the range 15–65 °C (see Figure S6), probably as a result of the overlapping of the contribution of the sol–gel temperature of each phospholipid-forming vesicles. Despite the fact that the vesicles were dispersed in a different solvent system and at a different concentration, the spectrum in Figure S6 shows the features typical of an unloaded liposomal formulation obtained from industrial scale (placebo, see [21]). This indicates that in the vesicles observed in Figure 3c,d, the lipids were organized in the membrane in a similar manner as in liposomes.

## 3. Discussion

In order to correlate the liposome size with the experimental conditions, the thermodynamics and kinetics of the liposome assembly process need to be discussed.

Liposomes are hypothesized to form in the MHF devices because the polarity of the lipid solvent increases during the mixing of alcohol with the aqueous buffer. Lipids become progressively less soluble in the mixture and self-assemble into planar lipid bilayers discs. During their growth, the discs begin to bend to reduce the surface area of the hydrophobic chains exposed to the polar solvent around the perimeter of the discs. Afterwards, the discs close into spherical vesicles, with the bilayer separating an aqueous interior from an aqueous exterior [22].

Liposome membranes are characterized by a phase transition temperature (Tc) below which the bilayer is in a more structured and rigid gel phase and above which the bilayer is in a more fluid and liquid crystalline phase. Tc increases with acyl chain length for saturated phospholipids. For liposomes formed from pure HSPC and pure mPEG2000-DSPE, Tc are 53 °C [23] and 12.8 °C [24], respectively. The addition of CHOL to phospholipids increases the stiffness of the bilayer and makes the elasticity modulus dependent on the temperature even at 10–15 °C above Tc [22].

Considering the working conditions herein used, liposomes were likely formed approximately or above Tc; this increased the mobility of the lipids bilayer and promoted the formation of small liposomes.

On the basis of the “non-equilibrium bilayer planar fragment model”, the liposome size is dependent on the kinetic growth rate of the bilayer planar discs and on the rate the discs bend and close into spherical vesicles.

The growth of the bilayer is due to the assembly of lipids solubilized in the alcohol/water mixture as well as to the aggregation of discs during the flow along the mixing channel length. The rate coefficient for the coalescence K(T) is temperature- and lipids concentration-dependent but it is not directly related to the flow conditions and geometric constraints. It is inversely proportional to the square of ξ, the distance over which coalescence can happen. A decrease in lipids concentration determines an increase in ξ, with a strong decrease of K(T). Therefore, at low lipids concentrations small diameter discs were formed, which led to the assembly of smaller size liposomes (as shown in Figure 4).

The rate at which the discs close into spherical vesicles is expressed by the characteristic closure time τ_c_, which decreases exponentially as the energy of curvature increases, as described by the Equation (1):

(1) $$\tau_{c}\left(T\right)=\tau_{z}\left(T\right)\exp\left[\frac{8\pi \tilde{\kappa }\left(T\right)}{\mathrm{KT}}{\left(1-\frac{V_{f}\left(T\right)}{2}\right)}^{2}\right]$$

where $\tilde{\kappa }$ is the effective bending elasticity modulus of the membrane, $\tau_{Z}\left(T\right)$ is related to the rotational relaxation time of a disc, and V_f_(T) represents the vesiculation index which is large for less stabilized discs. Ethanol acts as a surfactant in lipids assembly, inducing a stabilization effect of the discs.

At high FRRs, the magnitude of the shear stresses applied to the liposomes as they self-assemble increases. This effect produces thinner ethanol laminas and a smaller ethanol distribution, as shown in Figure 2 and discussed above. Therefore, at high FRRs, small liposomes were formed primarily, as discs had a limited time to grow, and the closure time was short. Conversely, larger liposomes were developed at lower FRRs, because the alcohol was depleted more slowly and the bilayers had more time to grow and stabilize.

In a previous work, by using similar flow conditions and 45° chips [11], phosphatidylcholine-based liposomes showed dimensions well below 100 nm. In the current work, the complexity of the lipid mixture probably introduced a higher rigidity of the bilayer [22]. The shear stress, applied by varying the FRRs, was not enough to destabilize the lipid discs and increase the V_f_. Hence, the V_f_ increased at high FRRs, but it was negligible compared with $\tilde{k}$ in Equation (1).

Zhigaltsev et al. have recently reported the production of nanoliposomal systems of different compositions using a staggered herringbone micromixer (SHM) [25]. Their results support our conclusions, since, by optimizing the preparation process with a different microfluidic method, they produced HSPC/mPEG200-DSPC/CHOL liposomes (56.2/38.5/5.3 mol/mol) of similar size (Z-average of 92.0 ± 5.5 nm) and polydispersity (0.15 ± 0.02). 

As a further consideration, the shear stress, which increases at high FRRs and promotes the assembly of small liposomes, can be enhanced by changing the inlet geometry. As demonstrated above, the injection of the alcoholic lipids mixture and of the aqueous buffering solution according to the 90° geometry improved the mixing rate. The thinner ethanol laminas and distributions generated in the focusing region, as well as the simulations of ethanol diffusion along the channel length indicated a more efficient mixing in the 90° chips. Smaller and size-monodisperse liposomes were produced within this geometry. Unfortunately, changes in geometry while keeping the FRR constant resulted in liposomes of: (i) smaller MDH but higher PDI in the 45° chips; and (ii) larger sizes with lower PDI in the 90° chips. Such outcomes strengthen our hypothesis that the characteristic lipid composition of the liposomes determines a high bending elasticity modulus, which dominates during the assembly process; this renders vesicle formation independent of the employed FRRs and the geometric constraints originally imposed to increase the shear stress and the mixing efficiency.

## 4. Materials and Methods 

### 4.1. Materials

Glass substrates were purchased from Telic Company (Valencia, CA, USA), isopropyl alcohol (IPA), acetone, sucrose, PBS, NaOH, HCl, NH_4_F, HF from Aldrich (Milan, Italy), the photoresist (AZ 9260) from MicroChemicals (Ulm, Germany), Chromium etchant solution (Poletto Aldo s.r.l., Noventa di Piave, Venice, Italy), Tubings (Tub FEP Blu 1/32 × 0.09) from IDEX_HS (IDEX Corporation, Lake Forest, Illinois, USA), TS10 glue from THORLABS (Newton, NJ, USA), filters with 0.22 μm (sterile-EO) pore size from Sartorius Stedim (Germany). The powders of hydrogenated soy phosphatidylcholine, HSPC, *N*-(carbonyl-methoxypolyethylene glycol 2000)-1,2-distearoyl-sn-glycero-3-phosphoethanolamine (mPEG-2000-DSPE), and cholesterol (CHOL) were provided by Janssen Pharmaceutical Company of Johnson & Johnson (Latina, Italy).

### 4.2. Fabrication of MHF Chips 

The glass substrates were cleaned with acetone and IPA. The designed patterns (shown in Figure 1) with microchannels of nominal width of 42 μm for the center inlet channel, 65 μm for the oblique side and mixing channel in the 45° chips, and of 36 μm for all the microchannels in the 90° chips were transferred from a photomask (J.D. Photo-tools Ltd., Oldham, Lancashire, UK) to the photoresist layer lying on the glass substrate via UV light exposure. The photoresist was then developed, followed by the removal of the exposed chromium layer using the etchant solution, thus revealing the microchannels onto the glass substrate itself. Exposed glasses were then wet etched with a buffered oxide etchant (BOE, composed by HF (48%): NH_4_F (1.38 M aqueous solution): HCl (38%) 1:41:1) solution prepared as reported in ref [16]. The wet etching procedure was performed by using a microwave reactor system, operating at a frequency of 50 GHz (Anton Paar Multiwave 3000, Labservice Analytica s.r.l., Anzola Emilia, Bologna, Italy). Microchannels with a depth h of about 100 µm (Large chips labelled as “L”), 50 µm (Medium chips labelled as “M”), and 25 µm (Small chips labelled as “L”) (as indicated in Table S1) were produced. Next, inlet and outlet holes were drilled on the etched substrates before sealing the microchannels by thermal bonding. Finally, the capillary tubes were glued to the inlet and outlet holes.

### 4.3. Set Up for Liposome Production in Flow

The set up optimized for liposome in-flow production at a fixed temperature consisted of four components (see Figure S1): (1) a single channel syringe pump (NE-1000 Multi-Phaser, New Era Pump Systems Inc., Farmingdale, NY USA) equipped with a tape heater (Syringe Heater, KF Technology, Rome, Italy) set at 55 °C for liposome injection; (2) a syringe pump with two channels (Ugo Basile, Biological Research Apparatus, model KDS270) for (NH_4_)_2_SO_4_ aqueous solution; (3) an optical microscope with a high resolution camera (Nikon Eclipse Ti); and (4) a temperature-controlled optical stage to allow facile visualization of the laminar flow during heating at 75 °C. The chips were connected to the syringe pumps and fixed on the microscope heating stage; the liposome solutions were collected from the outlet in a vial. The FRR used for the 45° chips were: 5, 10, 30, 50, 70, and 100 with Q_t_ of 110, 105, 120, 100, 140, and 100 µL/min. The FRR used for the 90° chips were: 1, 2, 3, 5, and 6 with Q_t_ of 54, 90, 84, 198, and 156 µL/min. 

### 4.4. Preparation of the Solutions

Lipids were combined in 56.2/38.5/5.3 mol/mol of HSPC/mPEG-DSPE/CHOL, as required for injectable liposomes. A solution of 90 mg/mL (mg of total lipids/mL of EtOH) was produced by stirring for 35 min on a hot plate at 60–65 °C in a closed vial; similarly, two more solutions of 9 mg/mL and 0.9 mg/mL were prepared. A 250 mM solution of (NH_4_)_2_SO_4_ was prepared in 18.2 MΩ·cm^−1^ water and filtered through 0.45 μm pore sized filters to prevent particulate contamination and clogging of the microfluidic device.

### 4.5. Dynamic Light Scattering (DLS)

The structural characterization of the vesicles (Z-average diameter of Mean Hydrodynamic Diameter, and polydispersity index) was evaluated by DLS using a Zetasizer Nano ZS90 (Malvern, PA, USA) equipped with a 4.0 mW He−Ne laser operating at 633 nm and with an avalanche photodiode detector. Measurements were made at 25 °C.

### 4.6. Transmission Electron Microscopy (TEM) 

The morphology of the vesicle dispersions was examined using transmission electron microscopy (TEM). Low-magnification TEM analyses were performed on a Jeol JEM-1011 electron microscope (Jeol Ltd., Akishima Tokyo, Japan) operating at 100 kV, equipped with a CCD camera ORIUS 831 from Gatan Inc. (Pleasanton, CA, USA). TEM samples were prepared by initially mixing dilute liposome dispersions, as eluted from the device, with a few microliters of 1% (*w*/*v*) osmium tetroxide aqueous solution, and then drop-casting them onto carbon-coated copper grids. Hence, each grid was rinsed twice in pure water and afterwards the deposited samples were completely dried at 60 °C for one night before examination.

### 4.7. Comsol Simulations

For numerical simulations, a two-dimensional model was implemented in Comsol Multiphysics to iteratively solve the continuity and Naviers–Stokes equations under laminar flow conditions. To evaluate the microfluidic flow, streamline distributions and fluid mixing within the MHF chips, i.e., the approach of Jahn et al. [19], was employed, modelling the system as if made of the same fluid with a different concentration of ethanol, which was allowed to diffuse. The dynamic viscosity could be related to the concentration gradient with the simplification that water and ethanol have similar viscosities in our system.

## 5. Conclusions

This study assessed the feasibility of the continuous-flow production of injectable liposomes characterized by a unique phospholipid mixture in MHF chips. 

A comparison of the mathematical model predictions and the experimentally measured values of the lamina thickness at different FRRs and inlet geometries was performed. In total agreement with the theory, the microsized dimensions of the lamina were found to decrease upon incrementing the FRRs and to be dependent on the chip inlet architecture. In the experiments, the impact of geometric constraints and lipids concentration on the liposome mean size and polydispersity at different FRRs was analyzed. Among these parameters, the key factor influencing the size of the liposomes and the PDI was clearly demonstrated to be the lipids concentration. The limited dependence on the other parameters was attributed to a predominance of the bending elasticity modulus over the vesiculation index, which made useless all the attempts to increase the applied shear stress (i.e., forcing the flow injection at 90° or increasing the FRRs). As a final outcome, liposomes of 100 nm average particle size and PDI ≤ 0.2, suitable for injectable solutions, were produced by using glass MHF chips. Injectable liposomes were also obtained using an SHM [25]. Although well performing, SHM microfluidic chips have a more complex design and require more expensive fabrication processes, and their scale-up is only possible by parallelization. In the case of MHF, no herringbone structures are needed, and a scale-up in the view of industrial production is not limited to parallelization as it was demonstrated to occur also by VFF. Therefore, for HSPC-based liposomes, MHF chips behaved as SHM chips with the advantage of a lower cost and easy scalability. In addition, the microfluidic approaches appear faster, cheaper, and more versatile compared to the current industrial production method based on ethanol injection [7].

## Figures and Tables

**Table 1:** Comparison between peaks areas of the simulations shown in Figure 2b,d and experimental lamina widths shown in the insets of Figure 1 and in Figure S3.

Chip Name	FRR	Peak Area (a.u.)	Lamina Width (μm)
45°	5	16.2	21 ± 2
45°	10	8.5	9 ± 3
45°	100	0.9	3 ± 1
90°	1	58.2	24 ± 3
90°	2	34.7	9 ± 2
90°	5	15.7	5 ± 3

## Supplementary Materials

The following are available online at http://www.mdpi.com/1996-1944/10/12/1411/s1. Scheme S1: Cross section of the mixing channel. w and h represent the width and the height, Table S1: Details about chip dimensions (width and height of the mixing microchannels) and internal volumes. “45° Chip” and “90° Chip” labels are related to Figure 1 and Scheme S1. “S”, “M” and “L” are referred to Small, Medium or Large chip dimensions, Table S2: MHD of the main peaks as determined from the DLS spectra of the samples produced at the lipids concentrations of 0.9 mg/mL and at different FRR using the 90°chip, Table S3: MHD of the main peaks as determined from the DLS spectra of the samples produced at different lipids concentration and FRR using the 45°chip, Figure S1: Set up for the measurements as described in experimental section, Figure S2: Ethanol distributions as calculated from the numerical simulations at the cross section indicated by the red arrows in Figure 2a and c, Figure S3: Experimental laminas inside “Large” 45° Chips at (a) FRR = 100, (b) FRR = 10 and (c) FRR = 5, Figure S4: Numerical simulations demonstrating the independence of the lamina peak area from Qt for 90° and 45° chips at FRR = 5, Figure S5: PDI values at different FRRs for 45° (black plots) and 90° chips (blue plot). The dependence on the lipids concentrations (90 mg/mL, 9 mg/mL and 0.9 mg/mL) is also shown for 45° chips, Figure S6: mDSC analysis of a sample produced in a 45°chip, using a lipids concentration of 9 mg/mL at FRR = 100. Calorimetric studies were carried out using a microDSC III (Setaram, France).
