# Supplementary material for: Continuous-Flow Production of Injectable Liposomes via a Microfluidic Approach
Source: Materials (Basel). 2017 Dec 10;10(12):1411. doi: 10.3390/ma10121411 (PMC5744346; doi:10.3390/ma10121411)
Supplement: Supplementary file 1 [file materials-10-01411-s001.doc]

Continuous flow production of injectable size liposomes via microfluidic approach

Alessandra Zizzari ^1,2^, Monica Bianco ^1^, Luigi Carbone ^1^, Elisabetta Perrone ^1^, Francesco Amato ^1^, Giuseppe Maruccio ^1,2^, Filippo Rendina ^3^ and ValentinaArima ^1^*

^1^ CNR NANOTEC - Institute of Nanotechnology, c/o Campus Ecotekne, University of Salento, Via Monteroni, 73100 Lecce, Italy

^2^ University of Salento, Department of Mathematics and Physics “E. De Giorgi” via Arnesano, 73100 Lecce, Italy

^3^ Janssen Pharmaceutical Company of Johnson & Johnson, via C. Janssen, Borgo S. Michele, Latina, Italy

* Correspondence: valentina.arima@nanotec.cnr.it; Tel.: +39-0832-319827

Received: 30 October 2017; Accepted: 07 December 2017; Published: 10 December 2017

**Scheme S1.** Cross section of the mixing channel. w and h represent the width and the height.

**
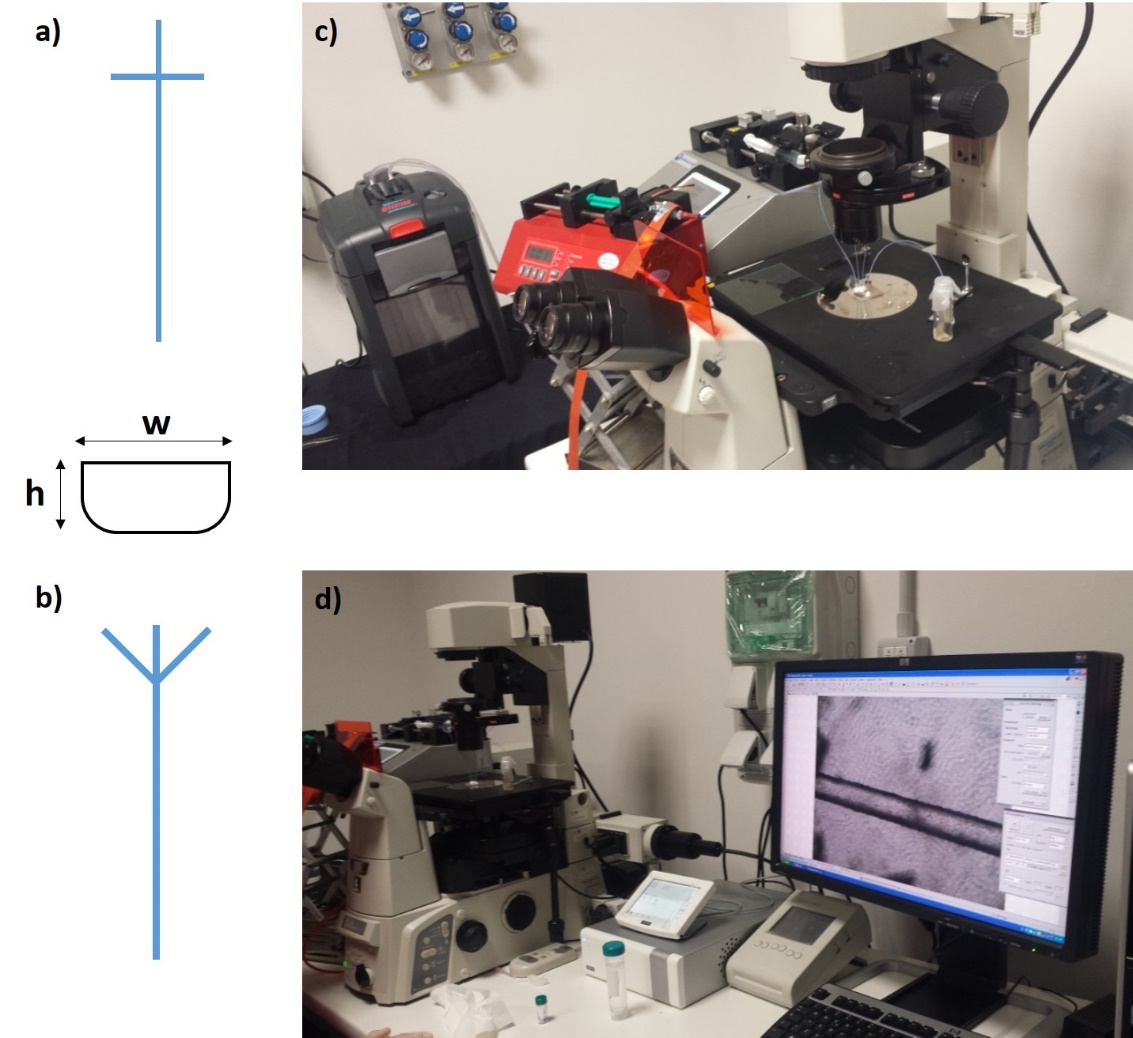
**

**Table S1.** Details about chip dimensions (width and height of the mixing microchannels) and internal volumes. “45° Chip” and “90° Chip” labels are related to Figure 1 and Scheme S1. “S”, “M” and “L” are referred to Small, Medium or Large chip dimensions.

|  | **45° Chip** | | | **90° Chip** | | |
| --- | --- | --- | --- | --- | --- | --- |
| **type** | S | M | L | S | M | L |
| **w** (μm) | 115 | 165 | 265 | 86 | 136 | 236 |
| **h** (μm) | 25 | 50 | 100 | 25 | 50 | 100 |
| **volume** (μm)^3^ | 7*10^7^ | 2*10^8^ | 6.6*10^8^ | 5.4*10^7^ | 1,7*10^8^ | 5.9*10^8^ |

**Figure S1.** Set up for the measurements as described in experimental section.


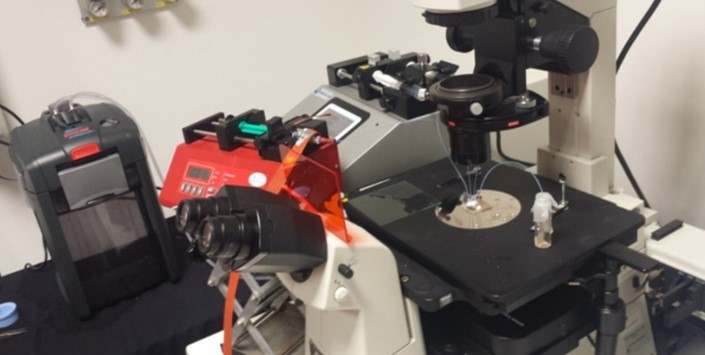


**Figure S2.** Ethanol distributions as calculated from the numerical simulations at the cross section indicated by the red arrows in Figure 2a and c.

**
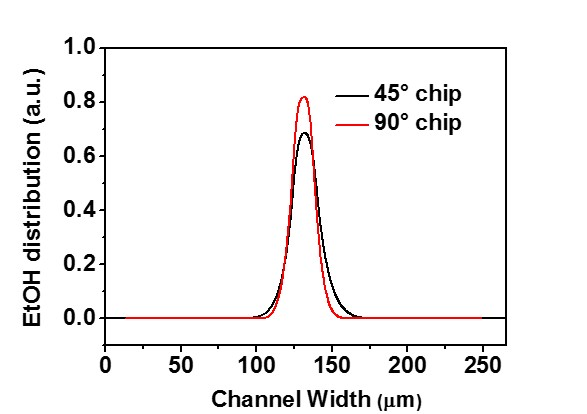
**

**Figure S3.** Experimental laminas inside “Large” 45° Chips at a) FRR= 100, b) FRR=10 and c) FRR=5.


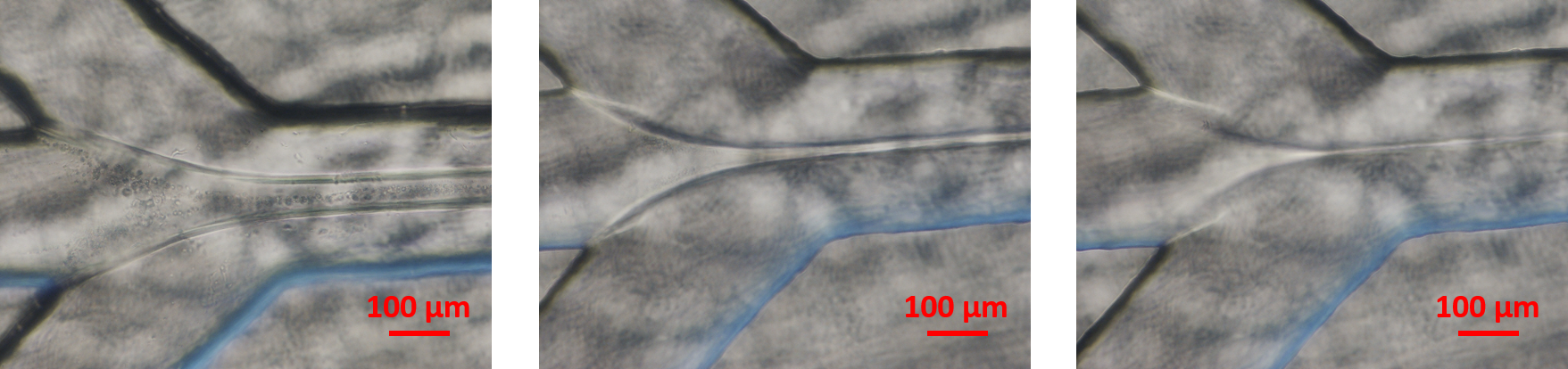


(a) (b) (c)

**Figure S4.** Numerical simulations demonstrating the independence of the lamina peak area from Q_t_ for 90° and 45° chips at FRR=5.

**
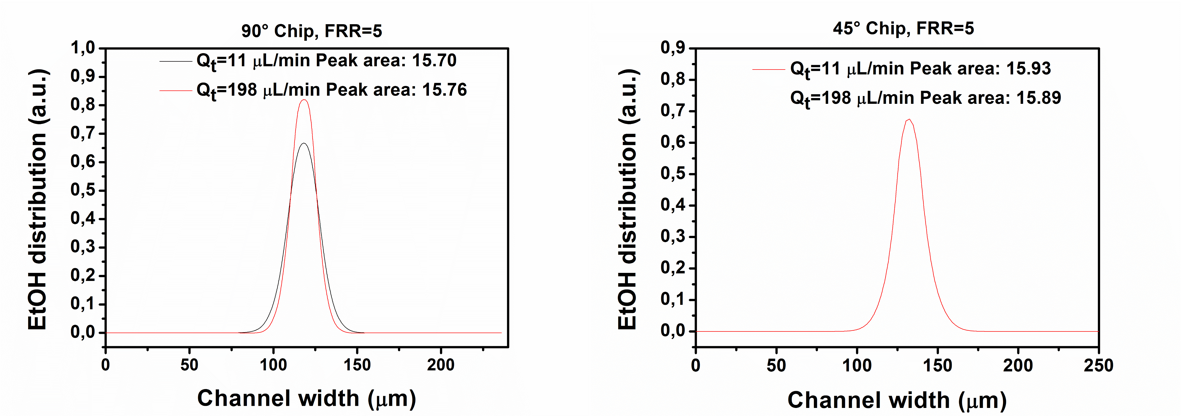
**

**Figure S5.** PDI values at different FRRs for 45° (black plots) and 90° chips (blue plot). The dependence on the lipids concentrations (90 mg/mL, 9 mg/mL and 0.9 mg/mL) is also shown for 45° chips.


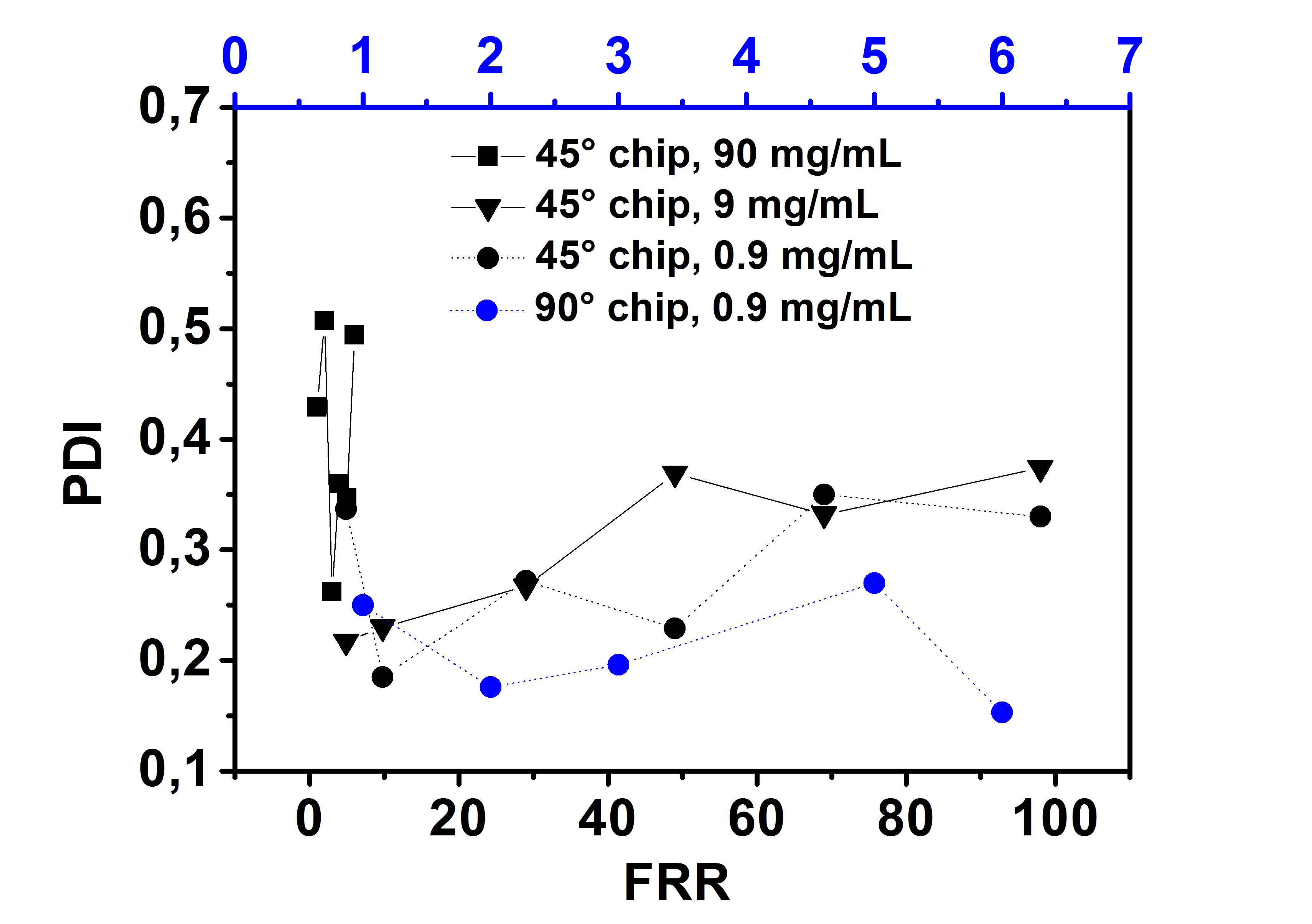


**Table S2. MHD of the main peaks as determined from the DLS spectra of the samples produced at the lipids concentrations of 0.9 mg/mL and at different FRR using the 90°chip.**

| **Concentration / FRR** | **0.9mg/mL (nm)** |
| --- | --- |
| **1** | 244±92 |
| **2** | 203±113 |
| **3** | 118±45 |
| **5** | 145±83 |
| **6** | 162±74 |

**Table S3. MHD of the main peaks as determined from the DLS spectra of the samples produced at different lipids concentration and FRR using the 45°chip.**

| **Concentration / FRR** | **90mg/mL (nm)** | **9mg/mL (nm)** | **0.9mg/mL (nm)** |
| --- | --- | --- | --- |
| **5** | 153±57; 675±389 | 204±87 | 70±21 |
| **10** | 347±283 | 388±199 | 94±29 |
| **30** | 381±295 | 231±45 | 118±61 |
| **50** | 238±138 | 163±139 | 93±37 |
| **70** | 247±147 | 73±22; 318±128 | 104±63 |
| **100** | 132±44; 414±155 | 249±154 | 106±59 |

**
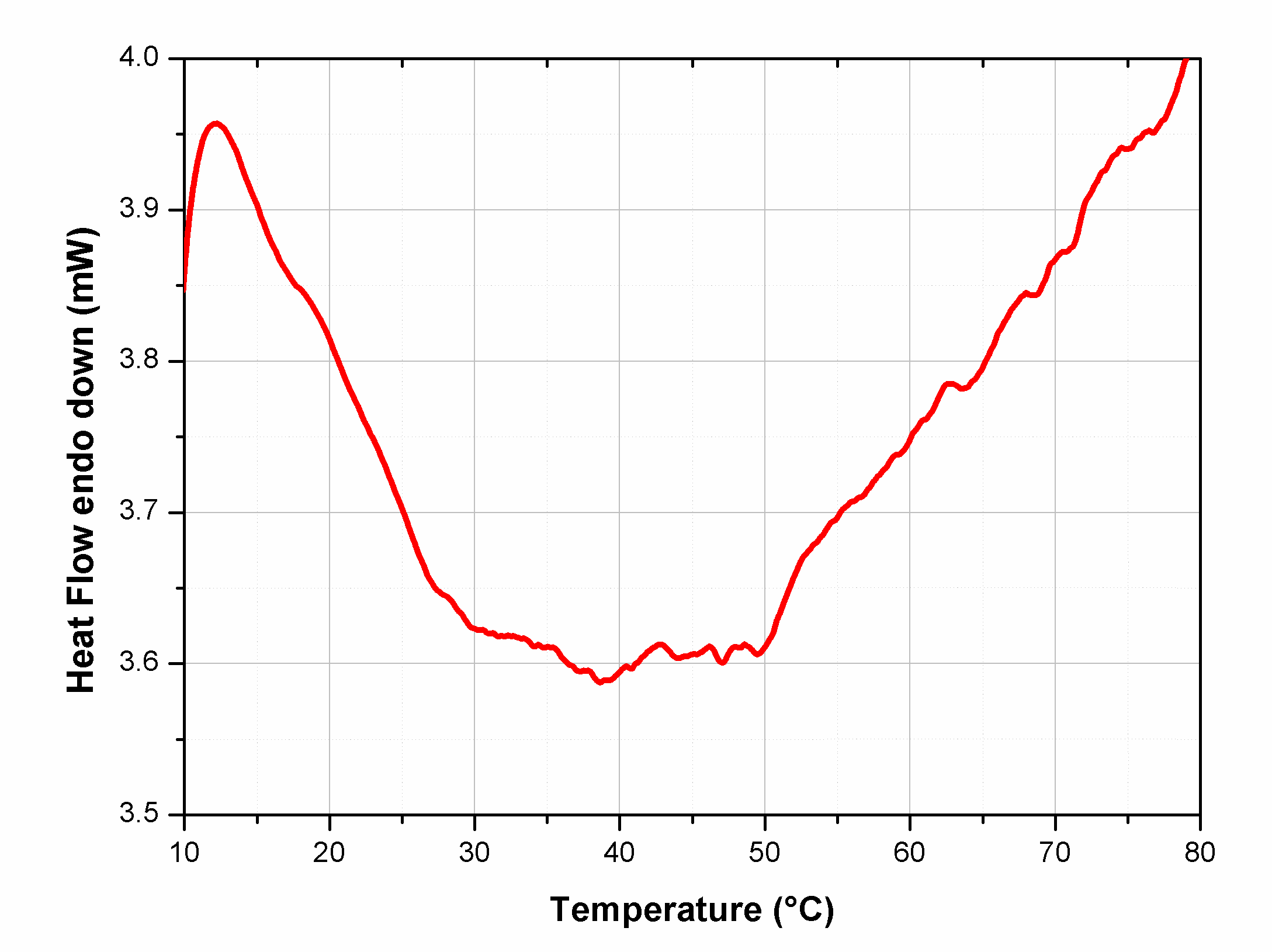
**

**Figure S6.** mDSC analysis of a sample produced in a 45°chip, using a lipids concentration of 9mg/mL at FRR=100. Calorimetric studies were carried out using a microDSC III (Setaram, France).
